# COVID-19 and domiciliary care utilisation: Evidence from the English Longitudinal Study of Ageing

**DOI:** 10.1016/j.jeoa.2025.100552

**Published:** 2025-06

**Authors:** Anastasia Arabadzhyan, Nikita Jacob, Panagiotis Kasteridis, Anne Mason, Nigel Rice

**Affiliations:** a Centre for Health Economics, University of York, UK; b Centre for Health Economics & Dept of Economics and Related Studies, University of York, UK

**Keywords:** Home care, COVID-19 pandemic, Inequality, Multinomial logit

## Abstract

The COVID-19 pandemic significantly affected global health and social care, leading to unmet needs, especially among vulnerable groups. Using data from the English Longitudinal Study of Ageing (ELSA), we investigate disruptions in home care for individuals over 50. We evaluate how the pandemic changed home care use at the extensive and intensive margins; the relative risk of reporting unmet need; and access to acute and primary care for different socio-demographic groups. We find decreases in home care use (extensive margin), mostly driven by informal care, which were partially offset by an increase in the amount of care received among those who were using home care during the pandemic (intensive margin). However, the relative risk of reporting unmet need rose, particularly among ethnic minorities, individuals with musculoskeletal and mental health conditions, and those not in work or retirement (due to long-term sickness or disability, home or family responsibilities, or unemployment). Individuals living alone and those aged 50–59 faced higher unmet needs for home care, but maintained primary care access as opposed to their counterparts. Our findings suggest that while aiming to protect the most vulnerable groups, pandemic containment policies negatively affected access to vital health and social care services, thereby increasing unmet care needs and exacerbating existing inequalities.

## Highlights

• We analyse home care use of those aged 50+ in England during the COVID-19 pandemic.
• We study heterogeneity in home care use changes at extensive and intensive margins.
• Informal care use fell, but care amounts rose for those who were receiving it.
• Unmet needs rose, especially in ethnic minorities, deepening existing inequalities.

## Introduction

Access to long-term care, also known as social care, becomes increasingly important as people age. As many older adults have to deal with chronic mental and physical health conditions, which affect their ability to perform daily tasks, social care services are key to ensure these individuals live as independently as possible, with high levels of well-being and life satisfaction.

Domiciliary care, or home care, is a vital element of social care.^1^ It offers a wide range of support services to individuals in their own homes, from healthcare monitoring to mobility assistance and meal preparation. This assistance may be provided formally, i.e. by a trained professional, or informally by a family member, friend, neighbour or volunteer. Utilisation of formal and informal care is determined by a wide range of factors. First, individual characteristics such as preferences, age, type and severity of the chronic illness or disability, drive the need and demand for care (Jarling et al., 2022). Second, the features of the social and institutional environment shape formal and informal care supply. For example, frequent contact with adult children is likely to lead to higher informal care use, while individuals with limited social support more often resort to formal care (Kjær and Siren, 2020). Formal care is typically used instead of, or in combination with, informal care in countries with more extensive welfare provisions (Suanet et al., 2012), while reductions in publicly-funded formal care was found to result in increased informal care utilisation (Zigante et al., 2021). This suggests that socio-economic inequality, by shaping the need for and ability to access home care, manifests itself in inequalities in formal and informal care use. For example, in the EU context, formal care was found to be concentrated in households with high socio-economic status, while informal care was more common among economically disadvantaged individuals (Lera et al., 2021). In light of this, it is important to ensure that the most vulnerable groups of society have timely access to alternative forms of care where there are disruptions to informal care provision, as switching to formal care might not be a feasible option for these individuals.

The COVID-19 pandemic in 2020 brought additional challenges for vulnerable and older adults. The introduction of lockdowns, social distancing, shielding, and other measures to contain the spread of the virus had a profound impact on both the provision of all types of care and the perceived effectiveness of care in meeting existing and newly emerging care needs. Older adults and those with chronic health conditions and weakened immune systems faced unique challenges trying to meet their care needs. Studies investigating the impact of the pandemic on formal and informal domiciliary care in various contexts generally find a decline in the reported use of formal care (Vislapuu et al., 2021, Tur-Sinai et al., 2021). Evidence on informal care is more mixed. In most countries, informal care provision and receipt appear to have been more resilient during the pandemic than that for formal care (Tur-Sinai et al., 2021). Some studies suggest an increased intensity of informal care provision and receipt (Vislapuu et al., 2021, Evandrou et al., 2020, Bergmann and Wagner, 2021), while others report no change (Rodrigues et al., 2021). In countries such as Denmark, the Netherlands and the US, however, the proportion of people with difficulties with activities of daily living who received informal care was lower during the pandemic than preceding it (Chen et al., 2022). Such differences are explained, in part, by a wide range of factors inherent to different study designs and settings, populations of interest (older or younger age groups, those living alone or with families, those with specific health conditions), use of different measures of informal care, lockdown rules and COVID-related policies put in place in different countries. Our study expands on the existing literature by investigating ways in which the COVID-19 pandemic impacted formal and informal care utilisation and subjective unmet care needs among older people in England. We make use of the English Longitudinal Study of Ageing (ELSA) due to its focus on community-dwelling respondents above 50 years of age, its longitudinal design, and the availability of data on use of care and whether respondents’ needs have been met. We make three contributions. First, we evaluate the impact of the pandemic on formal and informal home care utilisation across both the extensive and intensive margins.^2^ Existing studies on the impact of the pandemic on formal and informal care use (Vislapuu et al., 2021, Di Novi et al., 2023) employ utilisation measures which do not allow decomposition across both margins. However, this is an important step to understand the mechanism of the changes brought about by the pandemic. Certain subgroups may be less likely to use home care during the pandemic than before but conditional on receiving home care, they may receive more of it compared to the pre-pandemic period, or vice versa.

Second, we study how disruption in home care use manifests itself in respondents’ perception of how well care received met their needs before and during the pandemic. This is particularly important, since during the pandemic unmet healthcare needs were found to have a negative impact on older individuals’ health outcomes (Bergeot and Jusot, 2024).

Third, we explore whether disruptions to home care use observed during the pandemic were also present in primary and acute care settings. Specifically, we examine which population groups were affected by the pandemic in terms of cancellations of hospital operations and treatments and access to primary care. If substitution exists across care settings, we might expect to observe decreases in domiciliary care use being offset by increases in primary care use. The alternative is that the pandemic exacerbated existing inequalities in accessing care by limiting access to all care settings. For example, COVID-19 containment policies that aim to protect the most vulnerable groups may have both decreased home care provision and limited access to other health and care services for these same groups. Some evidence for the latter is provided by Topriceanu et al. (2021) who investigated the cancellation of surgical and medical appointments and concluded that the pandemic deepened health inequalities, with larger negative impacts on women, ethnic minorities and those with chronic conditions.

We find that community-dwelling respondents above 50 years of age experienced a decrease in the probability of receiving domiciliary care, mostly driven by a reduction in informal care use, during the pandemic. This effect was partially offset by a positive change on the intensive margin: on average, a majority of respondents using home care during the pandemic reported having received more care than pre-pandemic. However, we find an increase in the probability of reporting unmet need conditional on having care needs across many socio-economic and demographic sub-groups. Those most affected were ethnic minorities, individuals with certain health conditions (musculoskeletal and mental health), or permanently sick or disabled - groups more prone to unmet need (Spiers et al., 2022). Individuals living alone and the youngest age group (50–59 years), who have also been previously shown to be more susceptible to developing unmet need (Dunatchik et al., 2019), also saw a considerable increase in the risk of reporting unmet need for home care, but did not experience similar reductions in primary care access as their counterparts (i.e. those in multi-occupancy households and older cohorts). Taken as a whole, the results suggest that socio-demographic groups were impacted differently by the pandemic in their use of domiciliary care, unmet need and access to healthcare, potentially widening and entrenching pre-existing inequalities.

## Institutional background

Unlike healthcare provision, most social care services in England are not funded by the state. Social care is managed primarily by local authorities, which perform assessments of an individual’s needs and financial position to determine eligibility for access to publicly funded services and eligibility for financial assistance to cover the costs of social care. However, a very large number of individuals pay for social care out-of-pocket. Lyu et al. (2023) suggest that, for home care specifically, the proportion of people who cover all their related expenditures lies between 33% and 50%. Additionally, cuts in funding since 2010 have limited the range of services that can be provided. Coupled with an ageing population increasing the demand for social care, reductions in the availability of funded care provision results in unmet need. According to Bottery and Mallorie (2024), the gap between the number of individuals requesting social care support from local authorities has been consistently growing since 2015, while the number of individuals accessing this support has been falling — a trend which was exacerbated during the pandemic.

When formal care is not accessible, individuals tend to rely on informal care provision, as the two care types are often found to serve as substitutes for each other (Zigante et al., 2021, Saloniki et al., 2024). This increases the proportion of the population with caring responsibilities. In 2019, 19% of the English population older than 50 were informal carers — a two percentage point increase since 2015 (OECD, 2023, OECD, 2018). Given that informal care provision decreases caregivers’ labour market participation, this comes at a cost: Hu et al. (2023) estimate the economic cost of informal care in England in 2019 to be £54.2 billion, which was three times larger than expenditure on formal long-term care, and projected to rise to £101.4 billion by 2039.

## Data

ELSA is a biennial panel study of individuals aged 50 and above and their partners, living in English households, which has been running since 2002. The most recent wave preceding the pandemic was wave 9, the fieldwork for which was conducted during June 2018–July 2019. The survey provides detailed information on respondents’ socio-demographic characteristics, health status and behaviours, and healthcare utilisation. During the COVID-19 pandemic, instead of the regular data collection, two COVID-specific modules were run: the first during June–July 2020, and the second during November–December 2020. These modules do not include the full set of questions asked in the waves prior to the pandemic, but aim instead to assess the impact of COVID-19 and the related policies on health and wellbeing, and access to healthcare of ELSA respondents. Due to the restrictions in place during the pandemic, the COVID waves were conducted by online questionnaire or telephone interview. In total 7040 respondents were interviewed in the first wave and 6794 in the second, with about 17% of questionnaires completed via a telephone survey. To construct our estimation sample we use information from both COVID survey waves together with information from the last wave prior to the pandemic (wave 9). To ensure that the sample is representative and consistent throughout, and to mitigate possible effects of attrition, we apply longitudinal weights to both descriptive and statistical analyses. Since we rely on the information from the three consecutive waves (wave 9 and both waves 1 and 2 of the COVID period), we use the longitudinal population weights derived for core members who participated in all three waves. For more detail on the methodology behind the ELSA wave 9 and COVID waves weights see Addario et al. (2020).

Our core outcome variables of interest can be grouped into three categories: formal and informal domiciliary care utilisation; the change in the amount of care received conditional on using domiciliary care during the pandemic; and the respondent’s subjective evaluation of how well their needs were met by the domiciliary care they received. A summary of outcome variables and the survey questions used to construct these can be found in Table A.1 in the Appendix. For informal care in the pre-pandemic period, we rely on the set of questions in Module 4 (“Social care”) of the survey recording informal care utilisation as 1 if the respondent reported having received help with at least one of the activities of daily life in the past month from a relative, neighbour or friend, and zero otherwise.^3^ If help was received from a homecare worker, council handyman, cleaner, etc, this analogously is recorded as receiving formal care. For the pandemic period, formal and informal care use was inferred from the question “Over the past month have you received care at home?” and responses “Yes, formal (paid, provided from an agency)” and “Yes, informal (friend or relative)” respectively, for each wave. For each of the waves (pre-pandemic wave 9, and COVID waves 1 and 2) we constructed a categorical variable equal to 0 if no help/care was reported; 1 if only informal care was reported; 2 if only formal care use was reported; and 3 if use of both types of care was reported.

In addition to the home care use question, which reflects domiciliary care utilisation at the extensive margin, we look into whether those receiving care during the pandemic experienced an increase, a decrease, or no change in the amount of care, thus evaluating the change at the intensive margin. To this end, based on the question “Since the coronavirus outbreak started is the amount of care you receive...” we construct a categorical variable equal to 1 if a person responded “less than it was”; 2 if the response was “about the same”; and 3 if “more than it was”.^4^ Finally, we are interested in how well respondents’ needs are met when using domiciliary care. For the pre-pandemic period, this is measured through responses to the question “Thinking about all the help you receive, would you say that the help you receive (a) Meets your needs all the time, (b) usually meets your needs, (c) sometimes meets your needs or, (d) hardly ever meets your needs?”. Similarly, for the pandemic waves we use the question “Since the coronavirus outbreak started, have your care needs been met (a) All of the time (b) Most of the time (c) Some of the time (d) Hardly ever”. We construct a categorical variable equal to 1 if the respondent did not have care needs; 2 if, conditional on having care needs, they reported needs met all the time or usually/most of the time; and 3 if they reported needs being met only sometimes or hardly ever.^5^

In addition, we explore acute and primary care utilisation outcomes to investigate possible substitution or complementarity between health care and domiciliary care use. Acute and primary care utilisation includes two outcomes. The first is a binary indicator equal to 1 if the respondent answered “yes” to the question “Since the coronavirus outbreak, have you had a hospital operation or treatment cancelled?”, and 0 otherwise. This question is asked in both COVID waves. The second outcome relates to access to primary care. For the pre-pandemic period, this is measured via a binary variable equal to 1 if the person answered “yes” to the question “During the four weeks ending yesterday, did you talk to a family doctor (GP) about your own health either in person or by telephone?”, and 0 otherwise. For the first wave of the pandemic survey, we construct a similar indicator from the question “Have you been able to see or talk to a GP?”, conditional on answering “yes” to the question ”Since the coronavirus outbreak, have you wanted to see or talk to a GP?”. For those who were able to see or talk to the GP, the indicator is equal to 1, and 0 for everyone else.^6^

Our control variables include a large set of characteristics of respondents sourced from the pre-pandemic wave 9 survey. This includes age (as categories), sex, ethnicity, living in a rural area, job classification, employment status, number of household members, quintile of equalised income,^7^ presence of health conditions, and region of residence. Finally, we also include a control variable indicating whether a respondent had a positive COVID-19 test in waves 1 and 2 of the COVID survey.

Overall, the variables are very well-populated: the shares of observations with missing values range between 0 and 1.56%. The variables most affected by missing values are GP access before the pandemic (1.56% missing observations) and household income (1.36% missing observations). We note, however, that the occupation class variable has a considerable share (around 27%) of missing values. We therefore decided to retain the missing observations under the ‘unknown’ category of the occupation class variable. After cleaning the data and applying weights, our sample comprises 5033 individuals.

## Empirical approach

### Domiciliary care utilisation

We first analyse how the COVID-19 pandemic changed domiciliary care use across individuals with different socio-demographic and clinical characteristics. We focus on three categorical outcome variables: (1) domiciliary care use, with categories ‘none’, ‘informal care only’, ‘formal care only’, and ‘both’; (2) change in the amount of care received as compared to pre-pandemic, conditional on having used some type of domiciliary care during the pandemic, with categories ‘less than before’, ‘no change’, and ‘more than before’; (3) unmet need categorised as ‘no need’, ‘needs met always or usually’, and ‘needs met some of the time or hardly ever’. As an empirical strategy, we adopt a multinomial logistic regression models for outcomes (1) and (3). While more complex (e.g. two-stage) approaches could be conceived, we opted for a more parsimonious way of modelling the outcome variables which allows us to account for dependencies between not using home care (not having care needs) and using specific types of home care (having meeds met most of the time or some of the time). Outcome (2) is a set of ordered alternatives, we therefore adopt an ordered logistic regression to model it. For all models we evaluate predicted probabilities of each alternative for each of the individual characteristics. Accordingly, to investigate how the pandemic altered utilisation of domiciliary care at the extensive margin, we adopt the following specification:

(1) $$P\left(y_{it}=k\right)=\Lambda \left(\alpha +x_{i}^{\prime }\beta_{k}+\eta had\text{\_}covid_{it}+\gamma pandemic_{t}+x_{i}^{\prime }\times {\mathrm{pandemic}}_{t}\delta_{k}\right),$$

where $y_{it}$ takes value 0 if no domiciliary care was used, 1 if only informal care was used, 2 if only formal care was used, and 3 if both types of home care were used, thereby representing four alternatives (k={0,1,2,3}). Λ denotes the standard logistic cumulative distribution function. Since we observe the outcome over three periods (pre-pandemic, first, and second wave of the COVID ELSA survey), t={1,2,3}. The $pandemic_{t}$ variable takes value 1 if the observation belongs to one of the COVID waves, and $x_{i}^{\prime }\times {\mathrm{pandemic}}_{t}$ is the interaction between the vector of a respondent’s characteristics $x_{i}^{\prime }$ and the pandemic indicator.^8^ The $had\text{\_}covid_{it}$ variable takes value 1 if the individual reported having tested positive for COVID-19 in one of the COVID surveys. Note that although we include it in all the models, it cannot be considered a characteristic with respect to which a difference with the pandemic period is assessed, since no one had COVID before the pandemic. We then evaluate predicted probabilities of each alternative for each of the covariates, and analyse how much they differ when comparing the pre-pandemic and pandemic periods.

Next, we study the changes induced by the pandemic in home care utilisation at the intensive margin. This seeks to assess the extent that the amount of care received during the pandemic compares with that prior to the pandemic for respondents receiving domiciliary care. We specify and estimate a model of the form:

(2) $$P\left(y_{it}=k\right)=\Lambda \left(\alpha +x_{i}^{\prime }\beta_{k}+\eta had\text{\_}covid_{it}+\gamma wave_{t}\right),$$

where $y_{it}$ takes value 1 if the respondent reports having received less care, 2 if the same amount of care was received, and 3 if the care received during the pandemic was more than what the respondent used to receive before. Note that Eq. (2) differs from Eq. (1) in that the outcome is represented by three alternatives, therefore k={1,2,3}, and the question is only asked during the pandemic waves, so only two periods are observed (the two pandemic waves of the survey), hence wave is a dummy variable indicating an observation belonging to COVID wave 1. We then report predicted probabilities of receiving less, the same amount or more care for each individual characteristic.

Finally, we study whether the pandemic changed the extent to which domiciliary care received met respondents’ needs. We estimate a multinomial regression similar to Eq. (1), but the outcome $y_{it}$ now represents three alternatives (k={1,2,3}: not having care needs, needs met all the time or most of the time/usually, and care needs met hardly ever or sometimes. We present the results in terms of risk ratios (i.e. the relative risk of reporting unmet need conditional on having care needs) and the difference in risk ratios between pandemic and pre-pandemic periods. For each respondent group/characteristic j, the risk ratio for the pre-pandemic period is calculated as follows:

(3) $$RR_{j}^{pre}=\frac{\overset{ˆ}{P}\left(y=3|x_{j}=1,x_{-j},pandemic=0\right)}{\overset{ˆ}{P}\left(y=3|x_{j}=1,x_{-j},pandemic=0\right)+\overset{ˆ}{P}\left(y=2|x_{j}=1,x_{-j},pandemic=0\right)}$$

Here $\overset{ˆ}{P}\left(y=3|x_{j}=1,x_{-j},pandemic=0\right)$ is the predicted probability of reporting unmet need for socio-demographic group j (e.g. individuals of 50–59 years of age) pre-pandemic, while $\overset{ˆ}{P}\left(y=2|x_{j}=1,x_{-j},pandemic=0\right)$ is the corresponding probability of reporting needs reasonably met. The denominator, therefore, is the predicted probability of having care needs for group j in the pre-pandemic period. The analogous metric for the pandemic period is derived as follows:

(4) $$RR_{j}^{post}=\frac{\overset{ˆ}{P}\left(y=3|x_{j}=1,x_{-j},pandemic=1\right)}{\overset{ˆ}{P}\left(y=3|x_{j}=1,x_{-j},pandemic=1\right\}+\overset{ˆ}{P}\left\{y=2|x_{j}=1,x_{-j},pandemic=1\right)}$$

When reporting results, we present both risk ratios and their differences, $RR_{j}^{diff}=RR_{j}^{post}-RR_{j}^{pre}$.

### Cancelled treatment or procedures and access to GP services

To assess the degree of complementarity or substitutability between home care use and health care use we extend the above analysis to consider the use of several health care access variables. In particular, we exploit indicators available in ELSA for whether an operation or treatment was cancelled and whether an individual was able to access GP services.

To study the relationship between respondents’ characteristics and the probability of a cancelled treatment or procedure, we specify the following logistic regression model:

(5) $$P\left(y_{it}=1\right)=\Lambda \left(\alpha +x_{i}^{\prime }\beta +\eta had\text{\_}covid_{it}+\gamma wave_{t}\right),$$

where $y_{it}$ is a binary variable equal to 1 if individual i surveyed in COVID wave t (here t={1,2}) reported a cancellation of treatment or procedure since the start of the pandemic and 0 otherwise. $x_{i}$ is a vector of the respondent characteristics, and $wave_{t}$ is a dummy variable equal to 1 if the response relates to the second wave of the COVID ELSA survey, and 0 otherwise. Following estimation of Eq. (5) we calculate the predicted probability of a cancellation for each respondent, i, based on their set of characteristics, x.

We then study which groups of respondents were most affected by the pandemic in their access to GP services. For this, we are able to compare the use of GP services before and during the pandemic (note that this was not possible when looking at cancelled operations and/or treatments) and specify a logistic regression model of the form:^9^

(6) $$P\left(y_{it}=1\right)=\Lambda \left(\alpha +x_{i}^{\prime }\beta +\eta had\text{\_}covid_{it}+\gamma pandemic_{t}+x_{i}^{\prime }\times {\mathrm{pandemic}}_{t}\delta \right)$$

**Table 1:** Variable means (relative frequencies) for the weighted sample used for estimation.

Panel A: Outcome variables				
**Domiciliary care use**	Pre-pandemic N = 5,033	COVID W1 N = 5,031	COVID W2 N = 5,033	Total N = 15,097

Informal care only	0.112	0.065	0.065	0.081
Formal care only	0.016	0.011	0.016	0.014
Both	0.019	0.002	0.006	0.009

**Change in the amount of care received (care recipients only)**		COVID W1 N = 391	COVID W2 N = 383	Total N = 774

Less care than before		0.170	0.157	0.164
No change		0.543	0.638	0.590
More care than before		0.287	0.205	0.246

**Whether care needs are met**	Pre-pandemic N = 5,033	COVID W1 N = 5,030	COVID W2 N = 5,031	Total N = 15,094

All the time or usually	0.136	0.169	0.191	0.165
Sometimes or hardly ever	0.017	0.042	0.036	0.032
No needs	0.847	0.789	0.773	0.803

		COVID W1 N = 5,032	COVID W2 N = 5,030	Total N = 10,062

**Operation/treatment cancelled**		0.169	0.116	0.143

	Pre-pandemic N = 4,955	COVID W1 N = 5,033		Total N = 9,988

**GP access**	0.281	0.083		0.181

Panel B1: Control variables (measured at pandemic waves; N = 5,033)				

		COVID W1	COVID W2	
Had a positive COVID-19 test		0.004	0.015	

Panel B2: Control variables (measured at wave 9; N = 5,033)				

Variable	mean	Variable		mean

Age: 50–59	0.361	Rural		0.231
Age: 60–69	0.304	Ethnic minority		0.073
Age: 70–79	0.224			
Age: 80＋	0.111	*Health conditions*		
Job class.: Routine/manual	0.288	Ophthalmic condition		0.264
Job class.: Intermediate	0.193	Respiratory condition		0.136
Job class.: Managerial/admin	0.248	Musculoskeletal condition		0.310
Job class.: Unknown	0.271	Cancer		0.030
Employment status: Employee	0.346	Mental/behavioural disorder		0.081
Employment status: Self-employed	0.090	*Region*		0.052
Employment status: Retired	0.467	North East		0.134
Employment status: Sick/disabled	0.048	North West		0.134
Employment status: Other	0.049	Yorkshire and The Humber		0.100
Number of HH members: 1	0.222	East Midlands		0.090
Number of HH members: 2	0.523	West Midlands		0.105
Number of HH members: 3＋	0.255	East of England		0.115
Female	0.528	London		0.119
Income	640.3	South East		0.171
Equalised income	447.6	South West		0.114

Notes: Control variables are taken from wave 9 of the survey, except for testing positive for COVID-19. When entering the models, all control variables are binary indicators, hence the table does not report standard deviations as they are directly derived from the mean. Interpretation of results for categories with very low frequencies should be done with caution. The equalised income variable quintile dummies are used in regressions. For the purposes of descriptive statistics, the income variable has a standard deviation of 611 and ranges from 0 to 16,236; the corresponding values for equalised income are 395 and 0–10,824. Variables recorded pre-pandemic are taken from wave 9 of ELSA. The ‘Other’ employment status includes the unemployed and those looking after home or family. Longitudinal weights (linking the pre-pandemic wave and waves one and two of the survey) are applied throughout. W1 and W2 stand for waves 1 and 2 of the COVID surveys.

In Eq. (6) $y_{it}$ represents a binary variable equal to 1 if individual i surveyed in period t reported having accessed GP services (that is, either in the four weeks prior to the survey for the pre-pandemic period wave, or since the start of the pandemic for wave 1 of the COVID ELSA survey) and 0 otherwise. $pandemic_{t}$ is a dummy variable equal to 1 if the response was received during the pandemic (first wave of the COVID ELSA survey) and 0 otherwise; and $x_{i}^{\prime }\times {\mathrm{pandemic}}_{t}$ is the interaction between the respondents’ characteristics and the pandemic indicator. Having estimated this model, we obtain and compare predicted probabilities of accessing GP services before and during the pandemic.

All analyses are performed in Stata 18.

## Results

Table 1 presents descriptive statistics for our sample. For the utilisation of domiciliary care, informal care use decreased substantially, from 11% of respondents to about 7% during the pandemic. Interestingly, the share of individuals using only formal care did not change substantially, whereas the proportion of those using both types of home care fell from 2% to less than 1%. Around 60% of those who were receiving some form of domiciliary care during the pandemic reported no change in the amount of care received, 16%–17% experienced a decrease, and 29% (first wave) and 21% (second wave) of respondents reported an increase in the amount of care received. Finally, in the pandemic period we observe larger shares of respondents whose needs were met compared to those whose needs were only sometimes or never met by the care they received. However, this is in the context of a larger proportion of respondents having care needs during the pandemic (15% pre-pandemic and approximately 22% during the pandemic). If we consider how well the needs of those reporting having care needs were met before and during the pandemic, the share of respondents reporting sometimes or hardly ever increased from about 11% (pre-pandemic) to 20% in the first COVID survey wave, and to 16% in the second wave, indicating that a greater proportion of individuals experienced unmet needs.

With regards to acute and primary care outcomes, on average, about 17% and 11% of respondents reported having an operation or treatment cancelled during the first and the second survey waves respectively. The share of respondents who accessed GP services fell dramatically in the first wave of the pandemic.

In terms of respondent characteristics (our control variables), the age distribution is skewed towards younger age groups amongst the sample, 47% of individuals are retired, although a significant proportion are still employed (35%) or self-employed (9%). Most individuals live in two-person households (52%), but a substantial proportion live alone (22%). Slightly more than a half of the sample are female and nearly a quarter live in a rural area. The majority of respondents are of white ethnic group, with 7% being of other ethnicity. The average net weekly household income is approximately £640 with equalised income

of £448. Finally, approximately a third of respondents reported having a musculoskeletal condition, with ophthalmic (27%) and respiratory (15%) conditions also being relatively common.

**Fig. 1:**
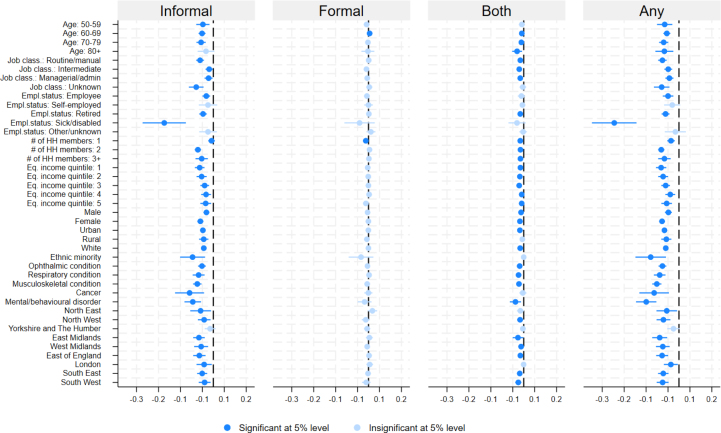
Differences in predicted probabilities of different types of domiciliary care use before and during the pandemic. Note: differences in conditional predicted probabilities derived from model (1) and their 95% CIs.

### Domiciliary care utilisation

Fig. 1 presents differences in the probability of domiciliary care utilisation before and during the pandemic, derived from estimation of Eq. (1). For each individual characteristic the figure depicts differences in the predicted probability of use for each of the three types of domiciliary care, as well as the probability of using any type of home care (the sum of the three), together with 95% confidence intervals for these differences. Original predicted probabilities for the outcomes are presented in Table A.2, and their differences in Table A.3, in the Appendix.^10^

In general, across most characteristics the probability of using informal care decreased in the pandemic period. This is not the case for the exclusive use of formal domiciliary care where the vast majority of characteristics do not display statistically significant differences in predicted probabilities, which is partly due to a low prevalence of an exclusive use of formal care in all periods. The decrease in the simultaneous use of both informal and formal care is possibly driven by the negative change in informal care use. It is also plausible that some of those individuals who previously would have used both types of care and lost access to informal care shifted to using only formal care following the onset of the pandemic, which would explain little to no change of sole formal care use.

From Fig. 1, the largest decreases in informal care use were observed for permanently sick or disabled respondents (−22 percentage points (pp)), ethnic minorities (−9.5 pp) and individuals with cancer (−10.8 pp) and mental health/behavioural disorders (−9.4 pp). If we consider the probability of using any type of domiciliary care (see column (4) in Table A.3), the same groups were among those most affected, but also including individuals with a musculoskeletal condition. We do not find pronounced gradients across age and rural–urban dimensions, but there appears to be a relationship with respect to equalised income (larger decreases for respondents with a lower income) and occupational class (those with unknown and routine/manual occupations experienced larger reductions in informal care than those with intermediate and managerial occupations).

Individuals living alone were the only group that experienced a statistically significant decrease in formal care use. Such individuals were amongst those most reliant on formal home care prior to the pandemic. Across geographical regions, the most affected area was East Midlands, while Yorkshire and The Humber was the least impacted region.

#### Amount of care used by care recipients

The results described above refer only to changes at the extensive margin, i.e. whether individuals were more or less likely to use home care during the pandemic than before. Table 2 presents results from estimation of Eq. (2). This analyses whether respondents who were receiving home care during the pandemic received more or less care compared to the pre-pandemic period. Table 1 shows that the majority of respondents reported no change in the amount of care received; approximately 24% reported receiving more care and about 16% reported having received less care than before. While this pattern generally holds across most of the individual respondent characteristics in which we are interested, some notable differences are apparent.

Some of the groups most affected by the negative change in home care use at the extensive margin, similarly experienced a negative impact on the intensive margin. Of those who were still able to access care during the pandemic, people from ethnic minority groups had the highest likelihood of reporting a decrease in care received (34%), which is three times as high as the likelihood of reporting an increase in care. Respondents of white ethnicity were 2.3 times less likely to report receiving less care than ethnic minority counterparts. As might be expected, individuals living alone had a higher likelihood of reporting a decrease in home care received than those living with another household member, also reinforcing the negative change observed at the extensive margin. In contrast, the self-employed, who appeared to be unaffected in terms of the probability of receiving home care, had the highest probability of reporting an increase in home care (47%). This might be due to the nature of work and flexibility to work from home, or spend more time at home. Residents in North East had the lowest predicted probability of reporting a decrease in care compared to other regions.

For individuals with a mental health condition or cancer the effect on home care use at the intensive margin might have partially counteracted the large decrease observed at the extensive margin. For individuals reporting a musculoskeletal condition, however, we observe a slightly higher than average probability of receiving less care during compared to before the pandemic.

**Table 2:** Predicted probabilities of change in domiciliary care received during the pandemic (N = 774)

	(1)		(2)		(3)	
	Less than before		Same		More than before	
	prob	s.e.	prob	s.e.	prob	s.e.
Age: 50–59	0.240**	0.095	0.599***	0.033	0.162**	0.071
Age: 60–69	0.155***	0.030	0.597***	0.024	0.248***	0.038
Age: 70–79	0.131***	0.024	0.583***	0.024	0.286***	0.035
Age: 80＋	0.154***	0.030	0.596***	0.024	0.250***	0.038
Job class.: Routine/manual	0.177***	0.026	0.595***	0.023	0.228***	0.026
Job class.: Intermediate	0.160***	0.027	0.590***	0.024	0.251***	0.032
Job class.: Managerial/admin	0.155***	0.031	0.587***	0.025	0.258***	0.039
Job class.: Unknown	0.156***	0.052	0.588***	0.036	0.256***	0.079
Employment status: Employee	0.270**	0.111	0.592***	0.047	0.138**	0.070
Employment status: Self-employed	0.062*	0.035	0.470***	0.108	0.468***	0.141
Employment status: Retired	0.155***	0.020	0.599***	0.023	0.245***	0.020
Employment status: Sick/disabled	0.147***	0.037	0.596***	0.031	0.258***	0.058
Employment status: Other	0.291***	0.111	0.583***	0.054	0.126**	0.061
Number of HH members: 1	0.174***	0.029	0.601***	0.023	0.225***	0.031
Number of HH members: 2	0.140***	0.018	0.586***	0.023	0.275***	0.024
Number of HH members: 3＋	0.192***	0.029	0.603***	0.023	0.205***	0.029
Equalised income quintile: 1 lowest	0.146***	0.024	0.584***	0.026	0.270***	0.035
Equalised income quintile: 2	0.172***	0.023	0.595***	0.024	0.233***	0.028
Equalised income quintile: 3	0.156***	0.031	0.589***	0.025	0.255***	0.040
Equalised income quintile: 4	0.213***	0.038	0.599***	0.024	0.187***	0.033
Equalised income quintile: 5 highest	0.136***	0.029	0.577***	0.027	0.287***	0.044
Male	0.172***	0.023	0.594***	0.023	0.234***	0.027
Female	0.159***	0.020	0.590***	0.023	0.251***	0.021
Urban	0.164***	0.017	0.592***	0.023	0.245***	0.019
Rural	0.164***	0.033	0.592***	0.023	0.244***	0.037
White	0.147***	0.017	0.601***	0.022	0.252***	0.018
Ethnic minority	0.338***	0.113	0.559***	0.068	0.104**	0.048
Had COVID	0.000	0.000	0.000	0.000	1.000***	0.000
*Health conditions*						
Ophthalmic condition	0.181***	0.021	0.598***	0.023	0.220***	0.020
Respiratory condition	0.175***	0.027	0.595***	0.023	0.229***	0.029
Musculoskeletal condition	0.187***	0.021	0.599***	0.023	0.214***	0.020
Cancer	0.179***	0.056	0.597***	0.026	0.224***	0.067
Mental/behavioural disorder	0.166***	0.039	0.593***	0.027	0.241***	0.054
*Region*						
North East	0.121***	0.034	0.570***	0.037	0.309***	0.063
North West	0.171***	0.033	0.599***	0.023	0.230***	0.037
Yorkshire and The Humber	0.144***	0.026	0.588***	0.025	0.268***	0.037
East Midlands	0.171***	0.047	0.599***	0.026	0.231***	0.057
West Midlands	0.211***	0.048	0.602***	0.024	0.187***	0.043
East of England	0.148***	0.028	0.590***	0.025	0.262***	0.038
London	0.179***	0.040	0.600***	0.025	0.220***	0.048
South East	0.150***	0.030	0.591***	0.026	0.259***	0.043
South West	0.156***	0.043	0.594***	0.026	0.251***	0.055

The analysis is conducted on a sub-sample of individuals who reported having received home care during the pandemic. Standard errors for predicted probabilities in parentheses. Significance levels: *** p<0.10, ** p<0.05, * p<0.01.

Finally, an interesting pattern emerges across age groups. At the extensive margin we observe a negative change of similar magnitude for all age groups. However, there are stark differences across the intensive margins. For respondents aged 60 and above who were receiving care during the pandemic, the predicted probability of reporting an increase in care was about 1.7 times the probability of reporting a decrease in care. This contrasts with the youngest group (50–59), who had similar predicted probabilities of receiving more or less care, implying older age groups generally received more care conditional on receiving care.

#### Did care meet respondents needs?

Across the majority of characteristics examined, the share of respondents receiving home care reduced during the pandemic, but at the same time for those who were receiving care in the pandemic period, a larger proportion were likely to report an increase, rather than a decrease, in the amount of care received. Taking into account the differential effects across both the extensive and intensive margins, the overall impact of the pandemic on the change in domiciliary care utilisation is ambiguous. Further insight can be gained by considering how respondents perceived whether the care they received met their needs.

First, we note that the proportion of respondents who indicated having care needs was higher during the pandemic (see Table 1, Table A.4 in the Appendix). This is likely due to lockdown rules creating a need for additional assistance (e.g. groceries or medicines delivery, which a respondent might previously have done themselves). To enable comparisons between periods we estimate risk ratios — the probability of reporting unmet need (for needs being met only some of the time or hardly ever, conditional on having care needs) as per Eqs. (3), (4). These are presented in Table 3 for the pre-pandemic and pandemic periods in columns (1) and (2), followed by their difference in column (3). The larger the difference between the pandemic and pre-pandemic risk ratios, the more substantial the impact of the pandemic on reporting unmet need.

For the majority of characteristics, we observe an increase in the relative risk of reporting unmet need, although in many cases the change is not statistically significant. As shown above, for some characteristics (ethnicity, living alone, being 50–59 years of age) the effect at the extensive and intensive margin operate in the same (negative) direction, so we expect to see an increase in the relative risk of reporting unmet need among these categories. Indeed, ethnic minorities saw the largest (by almost 30 pp.) increase in the relative risk of reporting unmet need. Respondents aged 50–59 and those living in single-person households also experienced large and statistically significant increases in the likelihood of reporting unmet need. An interesting exception are employed individuals. While this group experienced a reduction in care at both the extensive and intensive margin, this was not reflected in their propensity to report unmet need, possibly due to them having a lower level and complexity of care needs in general.

**Table 3:**
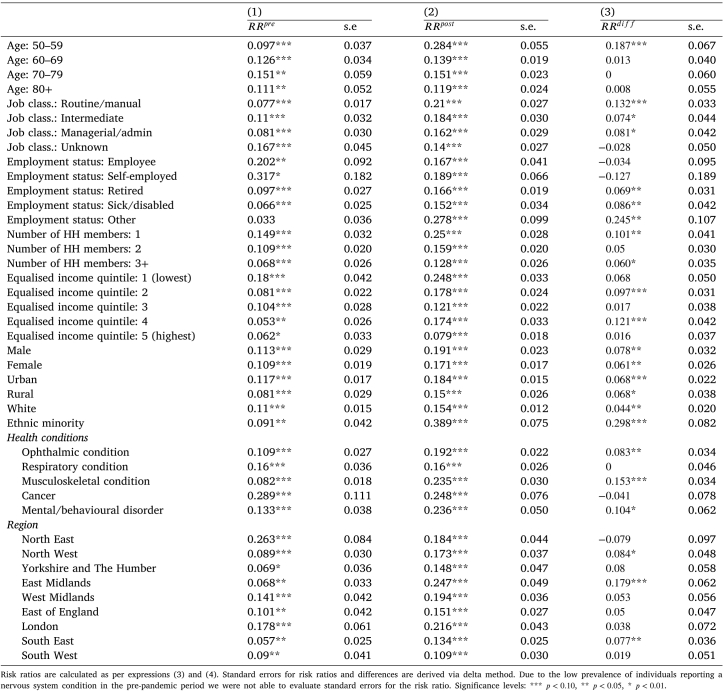
Risk ratios of reporting unmet need among those with care needs, before and during the pandemic (N = 15,094)

Among other substantially affected groups were individuals with ‘other’ employment status (unemployed and looking after home or family). Although this group did not see a statistically significant decrease at extensive margin, they were much more likely to report a decrease in the amount of care received. Those living in East Midlands saw some of the largest decreases at the extensive margin, and also experienced a large increase in unmet needs, possibly due to peculiarities of social care provision in the region. Similarly, individuals with a routine/manual occupation also experienced a considerable increase in the relative risk of reporting unmet need. However, this group was not among those with particularly high decreases in the probability of using domiciliary care or a negative change on the intensive margin.

Individuals with cancer did not report a change in unmet need. A potential explanation of this finding could be that starting from summer 2020 NHS England rolled out ‘COVID-friendly’ cancer treatments, allowing patients to receive some of the medications at home (NHS England, 2020). Although this was a replacement for secondary care services rather than a social care initiative, it may have led to some respondents perceive an increase in care received at home, leaving the relative risk of reporting unmet need unchanged.

However, respondents with a mental health/behavioural disorder or musculoskeletal condition experienced increases in the relative risk of reporting unmet need by 10.4 pp. and 15,3 pp. respectively. In addition, those with an ophthalmic condition had statistically significant increase in unmet need of 8.3 pp.

Taken together, the results suggest that even though in many cases respondents were likely to report an increase in the amount of care received during the pandemic, conditional on having received care in that period, the impact of the overall decrease in the probability of receiving any type of care during the pandemic led to an increase in unmet need.

**Table 4:**
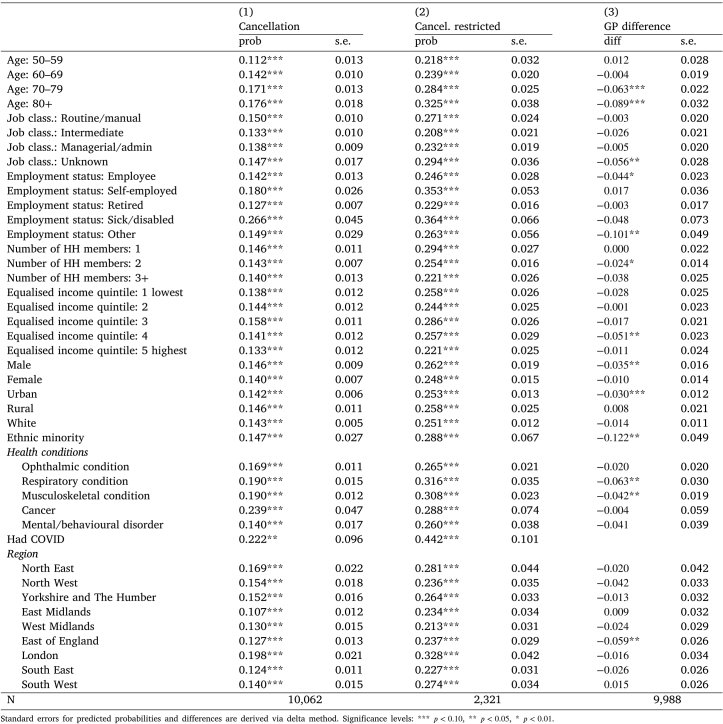
Predicted probabilities of cancelled treatment, and GP use before and during the pandemic.

### Cancelled treatment or procedures, and access to general practice

This section investigates whether the patterns revealed for home care use are repeated for healthcare utilisation. Table 4 provides predicted probabilities obtained from estimating models in Eqs. (5), (6). In the first column we report predicted probabilities and their standard errors of a treatment or procedure cancellation in the full sample. In the second column the results presented are obtained from a restricted sample, which includes only those individuals from the second wave of the COVID survey who reported having a treatment or procedure scheduled (it is not possible to identify those individuals in the first wave of the COVID survey). The final column represents the difference in the probability of accessing GP services prior to and during the pandemic. Our analysis of home care use revealed that individuals with certain socio-demographic and clinical characteristics were particularly affected by the pandemic in their access to home care and the likelihood of having unmet needs. Individuals aged 50–59 experienced a more pronounced disruption in domiciliary care use than older groups. However, Table 4 suggests that this group were less likely to experience a cancellation of treatment or procedure, nor a decrease in the probability of accessing GP services when compared to older cohorts. This is likely due to the older population being more vulnerable to COVID-19 and hence potentially benefiting from avoiding attending health care services. A similar pattern, possibly indicating some degree of substitution between home care and health care, emerges across regional area groups. East Midlands residents were most affected by the pandemic in the use of home care, but had the lowest probability of a cancellation and were generally not affected in terms of GP access. Individuals living alone experienced larger reductions in home care use and an increase in unmet need, but their ability to access GP services did not change during the pandemic compared to individuals living in larger households.

However, for some groups the pandemic had a pronounced negative impact on both domiciliary care and healthcare utilisation. Ethnic minorities experienced the largest decrease in home care use and the largest increase in unmet need, but also experienced a large reduction (about 12 pp. — the largest across all groups) in the likelihood of contacting a GP during the pandemic. Similarly, across employment groups, individuals in the ‘other’ category (includes unemployed, and looking after home or family) experienced a 10.1 pp. reduction in GP access, while permanently sick or disabled individuals saw were the most likely to be affected by cancellations.

Having a health condition was associated with a higher probability of a cancellation. This effect is plausibly due to individuals with health conditions being more vulnerable to COVID-19 compared to healthier counterparts. Those with a respiratory and musculoskeletal condition were most likely to have a treatment/procedure cancelled (with respective probabilities %31.6 and %30.8, conditional on treatment/procedure planned), and experienced the largest reductions accessing GP services (by 6.3 pp. and 4.2 pp. respectively). Overall, individuals with a musculoskeletal condition experienced a decrease in both home care use and healthcare use.

Taken as a whole, the results suggest that across some dimensions (age, number of household members, region) the pandemic had opposing effects, indicating a substitution, on home care and healthcare use. For other groups who experienced a decrease in domiciliary care use, and a resulting increase in unmet need (ethnic minorities, those with ‘other/unknown’ employment status, individuals with a musculoskeletal condition), their use of healthcare similarly decreased.

## Discussion and conclusions

Using the English Longitudinal Study of Ageing (ELSA), we study disruptions in home care for individuals over 50. Our work contributes to existing evidence by distinguishing changes in home care use at extensive and intensive margins, relating those changes to perceived unmet need, and investigating whether the pandemic led to similar changes in the healthcare use domain.

Our analyses indicate that the pandemic led to differential changes in home care use and unmet needs for social care across socio-economic and demographic groups within older adults in England. First, we evaluate the impact of the pandemic on the probability of using informal care (only), formal care (only), or both types of care across different characteristics of respondents. We interpret these results as revealing the impact on the extensive margin. Second, for those who reported having received home care during the pandemic, we investigate which groups were more likely to report a decrease or an increase in the amount of care received on the intensive margin. Third, we investigate variation in how the pandemic changed patterns of self-reported unmet need. Finally, we check whether the groups who were most negatively affected in terms of home care had substituted towards more healthcare service use (acute and primary care). Table 5 summarises our findings.

The pandemic led to decreases in the use of informal care and in the simultaneous use of both informal and formal care. The probability of informal care use decreased from approximately 11% to 7%, while the use of both types of care fell from 2% to about 0.4%. However, the propensity to use formal care alone remained fairly stable, at about 1.4%. We hypothesise that the decrease in informal care use was driven by lockdown, social distancing rules and shielding, when individuals were advised to minimise interactions with each other.

The negative impact on overall care received was, to an extent, mitigated across the intensive margin: among those who were receiving care during the pandemic, a higher proportion of respondents reported an increase in the amount of care received compared to those who reported a decrease. The overall net impact, however, across both margins is unclear.

**Table 5:** Changes in domiciliary care use, likelihood to report unmet home care need, and changes in acute and primary care access.

	Domiciliary care			Acute care	GP access
Variable	Extensive	Intensive	Likelihood to		
	margin change	margin change	report unmet need		
Age: 50–59	–	–	＋	–	No change
Age: 60–69	–	＋	No change	–	No change
Age: 70–79	–	＋	No change	–	–
Age: 80＋	–	＋	No change	–	–
Job class.: Routine/manual	–	＋	＋	–	No change
Job class.: Intermediate	–	＋	＋	–	No change
Job class.: Managerial/admin	–	＋	＋	–	No change
Job class.: Unknown	–	＋	No change	–	–
Employment status: Employee	–	–	No change	–	–
Employment status: Self-employed	No change	＋	No change	–	No change
Employment status: Retired	–	＋	＋	–	No change
Employment status: Sick/disabled	–	＋	＋	–	No change
Employment status: Other	No change	–	＋	–	–
Number of HH members: 1	–	＋	＋	–	No change
Number of HH members: 2	–	＋	No change	–	–
Number of HH members: 3＋	–	＋	＋	–	No change
Equalised income quintile: 1 (low)	–	＋	No change	–	No change
Equalised income quintile: 2	–	＋	＋	–	No change
Equalised income quintile: 3	–	＋	No change	–	No change
Equalised income quintile: 4	–	–	＋	–	–
Equalised income quintile: 5 (high)	–	＋	No change	–	No change
Male	–	＋	＋	–	–
Female	–	＋	＋	–	No change
Urban	–	＋	＋	–	–
Rural	–	＋	＋	–	No change
White	–	＋	＋	–	No change
Ethnic minority	–	–	＋	–	–
*Health conditions*					
Ophthalmic condition	–	＋	＋	–	No change
Respiratory condition	–	＋	No change	–	–
Musculoskeletal condition	–	＋	＋	–	–
Cancer	–	＋	No change	–	No change
Mental/behavioural disorder	–	＋	＋	–	No change
*Region*					
North East	–	＋	No change	–	No change
North West	–	＋	＋	–	No change
Yorkshire and The Humber	–	＋	No change	–	No change
East Midlands	–	＋	＋	–	No change
West Midlands	–	–	No change	–	No change
East of England	–	＋	No change	–	–
London	–	＋	No change	–	No change
South East	–	＋	＋	–	No change
South West	–	＋	No change	–	No change

Extensive margin change is inferred from column (4) in Table A.3 in the Appendix, and recorded as ‘No change’ if the difference is not statistically significant at 10% level. Intensive margin change is assumed to be positive if the predicted probability of reporting less care received is lower than that of reporting more care received, and negative if it is the other way around. Differences below 1 percentage point are considered as No change. The change in the likelihood of reporting unmet need is inferred from column (3) in Table 3 and recorded as ‘No change’ if the difference in risk ratios is not statistically significant at 10% level. Change in acute care is recorded as negative if the probability of cancellation is statistically significantly different from zero (column (1) in Table 4). GP access is inferred from column (4) in Table 4 and recorded as ‘No change’ if the difference is not statistically significant at 10% level.

It is notable that respondents were more likely to report having a need for home care during the pandemic (15% pre-pandemic and 22% during the pandemic).^11^ This is likely due to inaccessibility of other services (e.g. primary care) during lockdown periods, and needing assistance with activities of daily living that would otherwise expose an individual to the virus (e.g. grocery shopping). Unsurprisingly, higher levels of needs and difficulty in accessing domiciliary care resulted in higher levels of unmet need. Prior to the pandemic, the share of those needing home care whose needs were met only some of the time or hardly ever was 11%. This increased to about 18% in the pandemic period. It is also plausible that individuals started perceiving care received as less effective in meeting their needs, e.g. because communication became more difficult with face masks, or social distancing made respondents feel neglected. Since the unmet need measure we use is a self-reported one, it may suffer from this kind of bias. A more comprehensive understanding of need arguably requires both objective and subjective perspectives (Bradshaw, 1972). Pre-pandemic, in addition to subjective perception, needs were assessed through an interview, measured by difficulties with mobility, Activities of Daily Living (ADL) such as washing, dressing and feeding, and Instrumental Activities of Daily Living (IADLs) such as shopping or housework (Vlachantoni et al., 2011). However, in the COVID waves of the survey only the subjective measure was recorded. Although it is an imperfect measure, it appears to be valid as in many instances groups most affected in terms of receiving home care appear to be the ones more likely to report unmet need.

Our findings suggest that ethnic minorities were the group most affected by the pandemic. This group experienced the largest decrease in home care use across both the extensive and the intensive margins, and consequently were the group most likely to report unmet need. Importantly, the same group also experienced a pronounced decrease in primary care use. Accordingly, unmet need for home care was not compensated via other health care utilisation. This is in line with (Topriceanu et al., 2021) and suggests that the pandemic exacerbated existing inequalities, particularly by ethnicity, in access to care. Among other groups negatively impacted were some of the most vulnerable: those with certain health conditions (musculoskeletal and mental health); or the unemployed and those looking after home or family (the ‘other’ employment status group). These groups suffered substantial increases in the probability of needs becoming unmet. As with ethnic minority groups, these negative effects were not counterbalanced by greater access to primary care. The COVID-19 pandemic affected vulnerable groups disproportionately and this extends to the use of domiciliary care, a crucial component of support for elderly people. Our findings suggest that existing inequalities in the need for and access to care services that persisted prior to the pandemic where exacerbated leading to greater unmet needs and highlighting groups where health and care resources are most required.

Finally, the pandemic affected individuals across the age spectrum differently. The youngest age group (50–59) was among those experiencing the highest increase in the relative probability of reporting unmet need due to the pandemic. This was driven by negative changes to both the extensive and the intensive margins. However, being less vulnerable than their older counterparts, this group did not see a decrease in the likelihood of accessing GP services during the pandemic period. The older age groups, in contrast, did not see a statistically significant increase in the relative probability of reporting unmet need. Despite having experienced a decrease in overall care, and particularly informal care, these groups were among those most likely to report receiving more care, given care was received, than prior to the pandemic, which might have counteracted the extensive margin effect. This finding is in line with (Evandrou et al., 2020), and suggests that certain measures to assist the elderly population (such as volunteering to deliver grocery shopping) were effective.

Also of note are those least affected by the pandemic; those in work. Despite the negative impact of the pandemic on home care use at both margins, employees did not report an increase in unmet need. Finally, self-employed individuals did not see a substantial negative change to home care utilisation on either margin and their unmet need levels remained very similar to levels observed pre-pandemic. Moreover, this group did not see a decrease in the probability of accessing primary care.

Our study is subject to several limitations. First, the formal and informal care use and primary care access variables are constructed using survey questions with different formulations across waves (namely, the pre-pandemic and pandemic periods). While this may have led to a distortion in estimating absolute levels of domiciliary and primary care use, it is less likely to have affected our conclusions on about the heterogeneous impact across respondent characteristics, which is our main focus. Second, it is difficult to quantify the changes brought by the pandemic in the absence of data on the number of hours of care received, as well as the value/quality of care provided to respondents. While we are not able to properly measure these two aspects, we expect them to come together and manifest through unmet need. Finally, our analysis does not provide insight into the reasons why some socio-demographic groups were more affected than others. This is an extremely important question which warrants further research and would help to better understand, and therefore inform policies to reduce any long-term inequalities in formal and informal use of home care.

## CRediT authorship contribution statement

**Anastasia Arabadzhyan:** Writing – review & editing, Writing – original draft, Investigation, Methodology, Formal analysis, Conceptualization. **Nikita Jacob:** Writing – review & editing, Investigation, Methodology, Conceptualization. **Panagiotis Kasteridis:** Writing – review & editing, Investigation, Methodology, Conceptualization. **Anne Mason:** Writing – review & editing, Investigation, Methodology, Supervision, Funding acquisition, Conceptualization. **Nigel Rice:** Writing – review & editing, Investigation, Methodology, Supervision, Funding acquisition, Conceptualization.

## Declaration of competing interest

The authors declare that they have no known competing financial interests or personal relationships that could have appeared to influence the work reported in this paper.

## Figures and Tables

**Table A.1:** Construction of the outcome variables from the survey data.

Outcome variable	Values	Source question(s)	Survey wave
Domiciliary care pre-pandemic	1 if informal only (either response from 1 to 9 from Q1 and 96 for Q2), 2 if formal only (either response from 1 to 95 from Q2 and 96 for Q1), 3 if both (either response from 1 to 9 from Q1 and 1 to 95 for Q2), 0 otherwise	Q1 INFORMAL: In the last month, who has helped you with walking 100 yards/climbing stairs/walking across a room/getting in or out of bed/ using the toilet/ dressing/ bathing or showering/eating/ shopping for groceries/doing work around the house or garden/taking medications/managing money? Please only think about help received because of a physical, mental, emotional or memory problem. 1. Husband/ Wife/ Partner 2. Son (including step son, adopted son or son in law) 3. Daughter (including step daughter, adopted daughter or daughter in law) 4. Grandchild (including Great Grandchildren) 5. Sister (including step sister, adopted sister or sister in law) 6. Brother (including step brother, adopted brother or brother in law) 7. Other relative 8. Friend 9. Neighbour 96. None of these Q2 FORMAL: Now, please tell me about all of the people from this list who have helped you with walking 100 yards/climbing stairs/walking across a room/getting in or out of bed/ using the toilet/ dressing/ bathing or showering/eating/ shopping for groceries/doing work around the house or garden/taking medications/managing money in the last month? 1. Home care worker/ home help/ personal assistant 2. A member of the reablement/intermediate care staff team 3. Voluntary helper 4. Warden/Sheltered housing manager 5. Cleaner 6. Council’s handyman 7. Member of staff at the care/nursing home 95. Other - please specify 96. None of these	W9
Domiciliary care W1		Over the past month have you received care at home? 1. Yes, formal (paid, provided from an agency) 2. Yes, informal (friend or relative) 3. No	COVID W1
Domiciliary care W2			COVID W2

Change in the amount of care received W1	1 if less than before, 2 if No change, 3 if more than before	If used formal and/or informal care: Since the coronavirus outbreak started is the amount of care you receive…1. Less than it was 2. About the same 3. More than it was 4. no longer receive help	COVID W1
Change in the amount of care received W2			COVID W2

Whether care needs are met pre-pandemic	1 if no needs, 2 if all the time or usually, 3 if sometimes or hardly ever	Thinking about all the help you receive, would you say that the help 1. Meets your needs all the time 2. usually meets your needs 3. Sometimes meets your needs 4. Hardly ever meets your needs?	W9

Whether care needs are met W1	1 if no needs, 2 if all of the time or most of the time, 3 if some of the time or hardly ever	Since the coronavirus outbreak started, have your care needs been met…1. All of the time 2. Most of the time 3. Some of the time 4. Hardly ever 5. do not have any care needs	COVID W1
Whether care needs are met W2			COVID W2

Operation/treatment cancelled W1	1 if had a cancellation, 0 otherwise	Since the coronavirus outbreak, have you had a hospital operation or treatment cancelled? 1. Yes 2. No	COVID W1
Operation/treatment cancelled W2		Since the coronavirus outbreak, have you had a hospital operation or treatment cancelled? 1. Yes 2. No 3. I did not have a hospital operation or treatment booked	COVID W2

GP access pre-pandemic	1 if yes, 0 otherwise	During the four weeks ending yesterday, did you talk to a family doctor (GP) about your own health either in person or by telephone? 1. Yes 2. No	W9

GP access W1	1 if wanted to see a GP and was able to contact them, 0 otherwise	Since the coronavirus outbreak, have you wanted to see or talk to a GP? 1. Yes 2. No if yes: Have you been able to see or talk to a GP? 1. Yes 2. No 3. I did not attempt to contact them 4. I did not need to contact them	COVID W1

**Table A.2:** Predicted probabilities of home care use before and during the pandemic.

	Pre-pandemic			Pandemic		
	(1)	(2)	(3)	(4)	(5)	(6)
	Informal	Formal	Both	Informal	Formal	Both
	prob/s.e.	prob/s.e.	prob/s.e.	prob/s.e.	prob/s.e.	prob/s.e.
Age: 50–59	0.096***	0.016**	0.009	0.048***	0.008***	0.000***
	(0.016)	(0.007)	(0.007)	(0.010)	(0.003)	(0.000)
Age: 60–69	0.096***	0.003**	0.010***	0.044***	0.008***	0.002**
	(0.008)	(0.001)	(0.003)	(0.005)	(0.002)	(0.001)
Age: 70–79	0.122***	0.015***	0.015***	0.066***	0.012***	0.004***
	(0.011)	(0.005)	(0.003)	(0.007)	(0.003)	(0.002)
Age: 80＋	0.153***	0.041***	0.052***	0.119***	0.038***	0.021***
	(0.019)	(0.014)	(0.012)	(0.013)	(0.008)	(0.007)
Job class.: Routine/manual	0.125***	0.012***	0.019***	0.065***	0.013***	0.003***
	(0.009)	(0.004)	(0.004)	(0.005)	(0.002)	(0.001)
Job class.: Intermediate	0.090***	0.022***	0.023***	0.072***	0.012***	0.003**
	(0.009)	(0.006)	(0.005)	(0.007)	(0.003)	(0.001)
Job class.: Managerial/admin	0.077***	0.017***	0.020***	0.055***	0.011***	0.004***
	(0.008)	(0.004)	(0.005)	(0.006)	(0.002)	(0.001)
Job class.: Unknown	0.146***	0.017***	0.014	0.068***	0.020***	0.010***
	(0.018)	(0.005)	(0.009)	(0.011)	(0.005)	(0.004)
Employment status: Employee	0.049***	0.010	0.014*	0.017***	0.002*	0.003
	(0.010)	(0.007)	(0.008)	(0.005)	(0.001)	(0.003)
Employment status: Self-employed	0.057**	0.009	0.006	0.033***	0.009	0.000***
	(0.023)	(0.007)	(0.006)	(0.012)	(0.008)	(0.000)
Employment status: Retired	0.118***	0.014***	0.019***	0.071***	0.016***	0.003***
	(0.009)	(0.003)	(0.002)	(0.006)	(0.003)	(0.001)
Employment status: Sick/disabled	0.461***	0.080**	0.059***	0.237***	0.040***	0.029**
	(0.050)	(0.034)	(0.023)	(0.035)	(0.015)	(0.013)
Employment status: Other	0.075***	0.007	0.007	0.051***	0.018*	0.004
	(0.022)	(0.006)	(0.007)	(0.016)	(0.009)	(0.005)
Number of HH members: 1	0.074***	0.027***	0.020***	0.066***	0.014***	0.004***
	(0.008)	(0.005)	(0.004)	(0.006)	(0.003)	(0.001)
Number of HH members: 2	0.128***	0.009***	0.019***	0.058***	0.014***	0.004***
	(0.008)	(0.003)	(0.003)	(0.005)	(0.002)	(0.001)
Number of HH members: 3＋	0.137***	0.010*	0.016***	0.084***	0.012***	0.001
	(0.015)	(0.005)	(0.006)	(0.011)	(0.004)	(0.001)
Equalised income quintile: 1 (lowest)	0.123***	0.017***	0.017***	0.060***	0.014***	0.002**
	(0.012)	(0.006)	(0.005)	(0.007)	(0.003)	(0.001)
Equalised income quintile: 2	0.132***	0.013***	0.021***	0.078***	0.012***	0.003**
	(0.013)	(0.004)	(0.004)	(0.007)	(0.003)	(0.001)
Equalised income quintile: 3	0.108***	0.013***	0.026***	0.069***	0.013***	0.005***
	(0.010)	(0.004)	(0.005)	(0.007)	(0.003)	(0.002)
Equalised income quintile: 4	0.092***	0.017***	0.016***	0.059***	0.020***	0.007***
	(0.010)	(0.005)	(0.005)	(0.008)	(0.005)	(0.002)
Equalised income quintile: 5 (highest	0.081***	0.022***	0.013***	0.046***	0.010**	0.004*
	(0.012)	(0.007)	(0.004)	(0.008)	(0.004)	(0.002)
Male	0.094***	0.016***	0.015***	0.062***	0.012***	0.003***
	(0.007)	(0.004)	(0.004)	(0.005)	(0.002)	(0.001)
Female	0.126***	0.015***	0.022***	0.067***	0.015***	0.005***
	(0.007)	(0.003)	(0.003)	(0.005)	(0.002)	(0.001)
Urban	0.112***	0.015***	0.020***	0.065***	0.014***	0.003***
	(0.006)	(0.003)	(0.003)	(0.004)	(0.002)	(0.001)
Rural	0.110***	0.020***	0.016***	0.066***	0.013***	0.010***
	(0.011)	(0.006)	(0.004)	(0.007)	(0.003)	(0.003)
White	0.107***	0.014***	0.020***	0.064***	0.013***	0.004***
	(0.005)	(0.002)	(0.002)	(0.003)	(0.002)	(0.001)
Ethnic minority	0.180***	0.052*	0.003	0.086***	0.017*	0.003
	(0.033)	(0.027)	(0.004)	(0.017)	(0.010)	(0.003)
*Health conditions*						
Ophthalmic condition	0.125***	0.017***	0.023***	0.073***	0.012***	0.004***
	(0.009)	(0.004)	(0.004)	(0.006)	(0.002)	(0.001)
Respiratory condition	0.155***	0.013***	0.028***	0.088***	0.016***	0.005**
	(0.013)	(0.004)	(0.006)	(0.009)	(0.004)	(0.002)
Musculoskeletal condition	0.156***	0.023***	0.028***	0.083***	0.016***	0.006***
	(0.011)	(0.006)	(0.004)	(0.006)	(0.002)	(0.001)
Cancer	0.209***	0.018	0.011*	0.101***	0.017**	0.007
	(0.046)	(0.013)	(0.007)	(0.027)	(0.008)	(0.005)
Mental/behavioural disorder	0.163***	0.042***	0.042***	0.069***	0.025***	0.004*
	(0.022)	(0.016)	(0.013)	(0.012)	(0.007)	(0.002)
Had COVID				0.059	0.000*	0.029
				(0.041)	(0.000)	(0.020)
*Region*						
North East	0.129***	0.005	0.021**	0.071***	0.022**	0.006*
	(0.024)	(0.004)	(0.009)	(0.013)	(0.011)	(0.004)
North West	0.125***	0.023**	0.026***	0.083***	0.011***	0.009**
	(0.014)	(0.010)	(0.007)	(0.011)	(0.003)	(0.004)
Yorkshire and The Humber	0.083***	0.011	0.006**	0.068***	0.005**	0.002
	(0.012)	(0.007)	(0.003)	(0.010)	(0.002)	(0.001)
East Midlands	0.112***	0.016***	0.029**	0.047***	0.020***	0.003*
	(0.014)	(0.006)	(0.012)	(0.008)	(0.006)	(0.002)
West Midlands	0.120***	0.010*	0.013***	0.064***	0.004*	0.001
	(0.015)	(0.006)	(0.004)	(0.009)	(0.002)	(0.001)
East of England	0.117***	0.011**	0.019***	0.054***	0.014***	0.004
	(0.014)	(0.005)	(0.006)	(0.008)	(0.004)	(0.002)
London	0.122***	0.009	0.013**	0.080***	0.015***	0.012**
	(0.019)	(0.006)	(0.006)	(0.013)	(0.005)	(0.005)
South East	0.103***	0.021***	0.020***	0.053***	0.018***	0.002
	(0.013)	(0.005)	(0.005)	(0.007)	(0.005)	(0.001)
South West	0.103***	0.026***	0.027***	0.063***	0.016***	0.002*
	(0.014)	(0.009)	(0.007)	(0.010)	(0.005)	(0.001)

The ‘No care’ outcome probability is omitted from the table but can be derived as [1 - (columns (1)–(3))] and [1 - (columns (4)–(6)) ] for the pre-pandemic and pandemic periods respectively. The probability of using any type of home care is the sum of columns (1)–(3) and (4)–(6) for the pre-pandemic and pandemic, and can be found in Table A.2 in the Appendix (columns (1)–(2)). Standard errors for predicted probabilities in parentheses. Number of observations: 15,097. Significance levels: *** p<0.10, ** p<0.05, * p<0.01.

**Table A.3:**
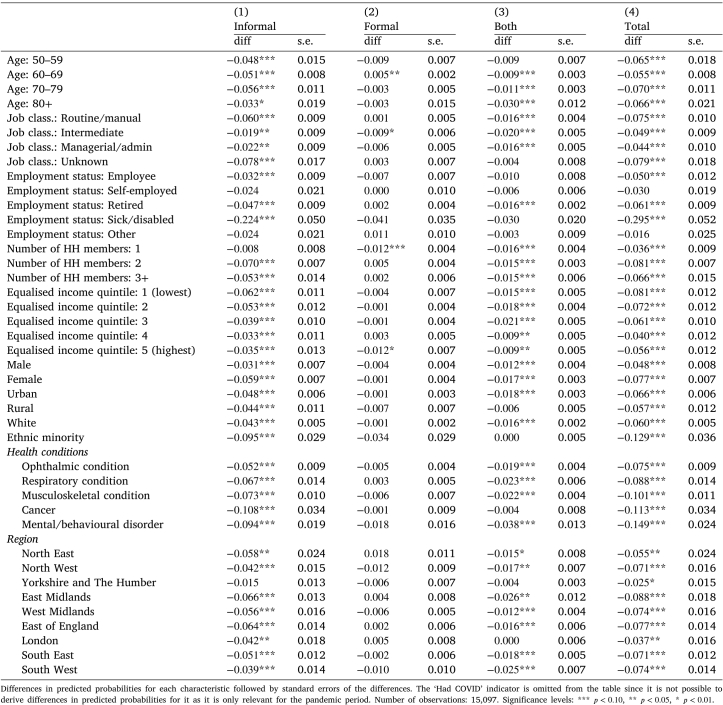
Differences in predicted probabilities of domiciliary care use.

**Table A.4:** Predicted probabilities of care needs being met before and during the pandemic (N = 15,094).

	Pre-pandemic			Pandemic		
	(1)	(2)	(3)	(4)	(5)	(6)
	No needs	Always or	Sometimes or	No needs	Always or	Sometimes or
		usually	hardly ever		usually	hardly ever
	prob/s.e.	prob/s.e.	prob/s.e.	prob/s.e.	prob/s.e.	prob/s.e.
Age: 50–59	0.869***	0.118***	0.013***	0.819***	0.129***	0.051***
	(0.020)	(0.019)	(0.005)	(0.016)	(0.013)	(0.012)
Age: 60–69	0.887***	0.098***	0.014***	0.798***	0.174***	0.028***
	(0.009)	(0.008)	(0.004)	(0.011)	(0.010)	(0.004)
Age: 70–79	0.843***	0.133***	0.024**	0.762***	0.202***	0.036***
	(0.012)	(0.011)	(0.010)	(0.013)	(0.013)	(0.006)
Age: 80＋	0.739***	0.232***	0.029**	0.652***	0.306***	0.042***
	(0.022)	(0.020)	(0.014)	(0.023)	(0.023)	(0.008)
Job class.: Routine/manual	0.839***	0.149***	0.012***	0.783***	0.172***	0.046***
	(0.010)	(0.010)	(0.003)	(0.011)	(0.009)	(0.007)
Job class.: Intermediate	0.861***	0.124***	0.015***	0.788***	0.173***	0.039***
	(0.010)	(0.010)	(0.005)	(0.011)	(0.011)	(0.007)
Job class.: Managerial/admin	0.880***	0.111***	0.010***	0.810***	0.159***	0.031***
	(0.010)	(0.009)	(0.004)	(0.010)	(0.009)	(0.006)
Job class.: Unknown	0.812***	0.156***	0.031***	0.740***	0.223***	0.036***
	(0.019)	(0.018)	(0.009)	(0.018)	(0.017)	(0.007)
Employment status: Employee	0.927***	0.058***	0.015*	0.828***	0.143***	0.029***
	(0.012)	(0.011)	(0.008)	(0.014)	(0.012)	(0.008)
Employment status: Self-employed	0.925***	0.051**	0.024	0.823***	0.144***	0.034**
	(0.024)	(0.020)	(0.017)	(0.022)	(0.020)	(0.013)
Employment status: Retired	0.841***	0.143***	0.015***	0.775***	0.188***	0.037***
	(0.010)	(0.010)	(0.005)	(0.010)	(0.009)	(0.005)
Employment status: Sick/disabled	0.378***	0.581***	0.041***	0.449***	0.467***	0.084***
	(0.052)	(0.053)	(0.015)	(0.048)	(0.047)	(0.019)
Employment status: Other	0.891***	0.106***	0.004	0.836***	0.118***	0.046**
	(0.027)	(0.027)	(0.004)	(0.026)	(0.023)	(0.019)
Number of HH members: 1	0.870***	0.111***	0.019***	0.790***	0.158***	0.053***
	(0.010)	(0.009)	(0.004)	(0.012)	(0.010)	(0.007)
Number of HH members: 2	0.839***	0.144***	0.018***	0.787***	0.179***	0.034***
	(0.008)	(0.007)	(0.003)	(0.008)	(0.007)	(0.005)
Number of HH members: 3＋	0.832***	0.157***	0.011**	0.756***	0.213***	0.031***
	(0.015)	(0.015)	(0.004)	(0.015)	(0.015)	(0.007)
Equalised income quintile: 1 (lowest)	0.839***	0.132***	0.029***	0.773***	0.171***	0.056***
	(0.014)	(0.012)	(0.008)	(0.014)	(0.012)	(0.009)
Equalised income quintile: 2	0.828***	0.158***	0.014***	0.772***	0.188***	0.041***
	(0.013)	(0.013)	(0.004)	(0.013)	(0.012)	(0.005)
Equalised income quintile: 3	0.844***	0.140***	0.016***	0.780***	0.193***	0.027***
	(0.011)	(0.010)	(0.005)	(0.013)	(0.012)	(0.005)
Equalised income quintile: 4	0.863***	0.129***	0.007**	0.806***	0.160***	0.034***
	(0.011)	(0.011)	(0.004)	(0.012)	(0.011)	(0.007)
Equalised income quintile: 5 (highest)	0.883***	0.109***	0.007*	0.787***	0.196***	0.017***
	(0.013)	(0.012)	(0.004)	(0.013)	(0.013)	(0.004)
Male	0.874***	0.112***	0.014***	0.775***	0.182***	0.043***
	(0.008)	(0.008)	(0.004)	(0.009)	(0.008)	(0.006)
Female	0.828***	0.153***	0.019***	0.785***	0.178***	0.037***
	(0.007)	(0.007)	(0.003)	(0.008)	(0.007)	(0.004)
Urban	0.849***	0.134***	0.018***	0.782***	0.178***	0.040***
	(0.006)	(0.006)	(0.003)	(0.006)	(0.006)	(0.004)
Rural	0.844***	0.144***	0.013***	0.777***	0.189***	0.033***
	(0.011)	(0.011)	(0.005)	(0.012)	(0.011)	(0.006)
White	0.853***	0.131***	0.016***	0.784***	0.183***	0.033***
	(0.005)	(0.005)	(0.002)	(0.006)	(0.006)	(0.003)
Ethnic minority	0.761***	0.217***	0.022**	0.754***	0.150***	0.096***
	(0.036)	(0.035)	(0.010)	(0.032)	(0.026)	(0.023)
*Health conditions*						
Ophthalmic condition	0.830***	0.151***	0.019***	0.760***	0.194***	0.046***
	(0.010)	(0.009)	(0.005)	(0.011)	(0.010)	(0.006)
Respiratory condition	0.796***	0.171***	0.033***	0.721***	0.235***	0.045***
	(0.014)	(0.013)	(0.008)	(0.015)	(0.015)	(0.008)
Musculoskeletal condition	0.787***	0.195***	0.018***	0.736***	0.202***	0.062***
	(0.012)	(0.012)	(0.004)	(0.012)	(0.010)	(0.009)
Cancer	0.762***	0.170***	0.069**	0.660***	0.256***	0.084***
	(0.042)	(0.032)	(0.033)	(0.044)	(0.038)	(0.030)
Mental/behavioural disorder	0.750***	0.217***	0.033***	0.729***	0.207***	0.064***
	(0.026)	(0.026)	(0.010)	(0.025)	(0.023)	(0.015)
Had COVID				0.698***	0.302***	0.000*
				(0.082)	(0.082)	(0.000)
*Region*						
North East	0.838***	0.119***	0.043**	0.766***	0.191***	0.043***
	(0.027)	(0.020)	(0.017)	(0.022)	(0.020)	(0.011)
North West	0.818***	0.165***	0.016***	0.747***	0.209***	0.044***
	(0.017)	(0.017)	(0.005)	(0.018)	(0.017)	(0.010)
Yorkshire and The Humber	0.892***	0.101***	0.007*	0.828***	0.146***	0.025***
	(0.014)	(0.014)	(0.004)	(0.015)	(0.013)	(0.009)
East Midlands	0.839***	0.150***	0.011**	0.781***	0.165***	0.054***
	(0.018)	(0.017)	(0.005)	(0.019)	(0.016)	(0.013)
West Midlands	0.841***	0.137***	0.022***	0.790***	0.169***	0.041***
	(0.016)	(0.017)	(0.007)	(0.017)	(0.015)	(0.008)
East of England	0.853***	0.132***	0.015**	0.800***	0.170***	0.030***
	(0.013)	(0.013)	(0.006)	(0.013)	(0.013)	(0.006)
London	0.853***	0.121***	0.026***	0.725***	0.215***	0.059***
	(0.019)	(0.018)	(0.010)	(0.021)	(0.020)	(0.013)
South East	0.849***	0.142***	0.009**	0.803***	0.171***	0.026***
	(0.013)	(0.013)	(0.004)	(0.013)	(0.013)	(0.005)
South West	0.844***	0.142***	0.014**	0.791***	0.186***	0.023***
	(0.015)	(0.015)	(0.007)	(0.017)	(0.017)	(0.006)

Standard errors for predicted probabilities in parentheses. Significance levels: *** p<0.10, ** p<0.05, * p<0.01.

**Table B.1:**
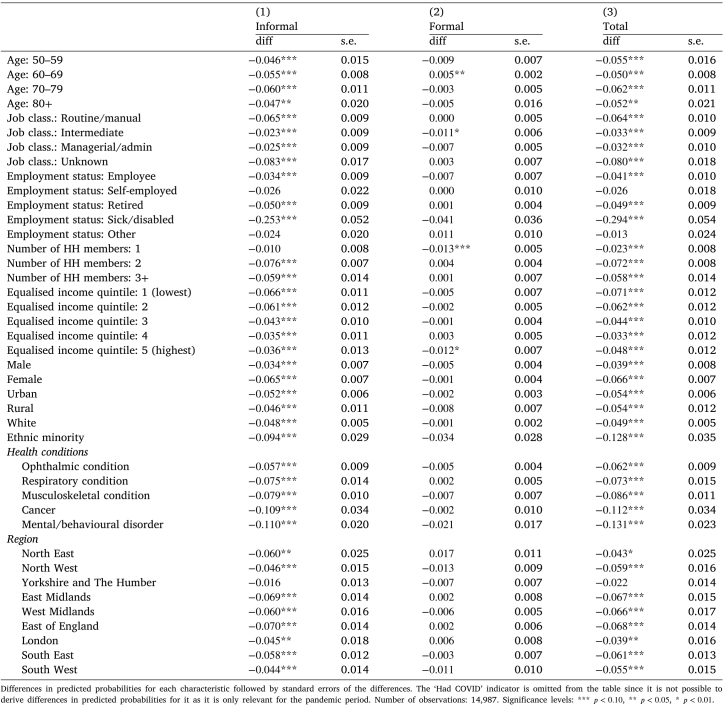
Differences in predicted probabilities of domiciliary care use.

**Table B.2:**
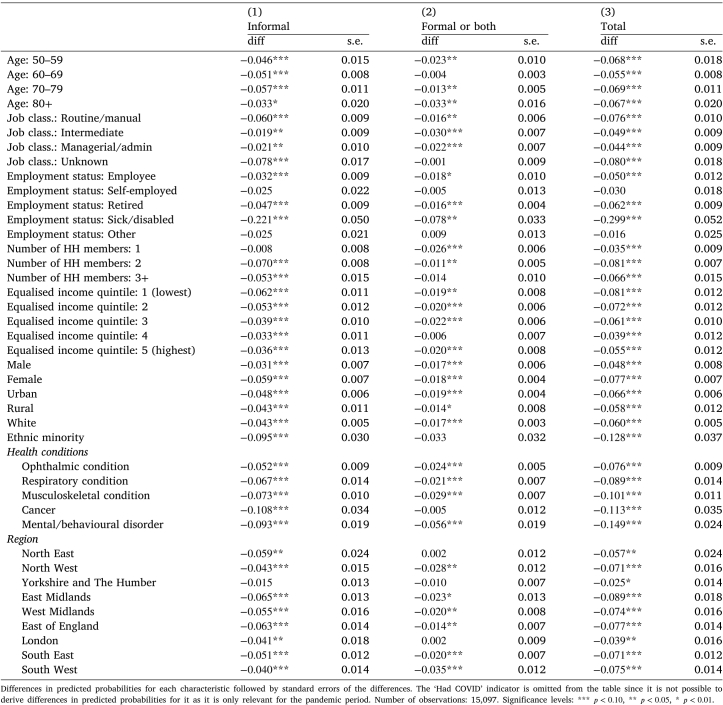
Differences in predicted probabilities of domiciliary care use.

## Appendix B

### Alternative specifications of the domiciliary care use model

Formal tests (Hausman and suest-based Hausman) suggest that the IIA (independence of irrelevant alternatives) assumption does not hold for model (1). This is due to inclusion of the last category, ‘both types of care’. If one type of home care becomes disproportionately more/less available, the independence between this type of care and the use of both types of care is violated. To understand how this impacts our results, we conducted two robustness checks. First, we dropped the ‘both types of care’ category and estimated the multinomial logistic regression with three categories in the outcome variable: no care, informal care only, formal care only. This model passes the IIA tests. The results are presented in Table B.1, and are very similar to our baseline findings quantified in Table A.3.

Another specification we consider involves re-categorisation of the outcome variable into ‘no care’, ‘informal care only’, ‘formal care or both types of care’. This also allows the model to meet the requirements of IIA tests. The results are presented in Table B.2, and are as expected. Specifically, analysing the differences in predicted probabilities of using various types of care before and during the pandemic, we see that the figures obtained for the new ‘formal care or both types of care’ category turned out to be a sum of those previously obtained for ‘formal care only’ and ‘both types of care’ (columns 3 and 5 in Table A.3), with very minor discrepancies. We therefore confirm that the results of our baseline model are very similar to those from alternative specifications which pass the formal IIA tests.

## Footnotes

1 Within the remit of this work, we use the terms ‘domiciliary care’ and ‘home care’ interchangeably and as an umbrella term for any type of care an individual receives at home.

2 Changes to the extensive margin refer to a change in the likelihood of using domiciliary care, while the intensive margin reflects whether conditional on receiving care an individual is using more or less care.

3 Activities of daily life include walking, going up and down the stairs, getting in and out of bed, dressing, using the toilet and bathing/showering, eating, housework or gardening tasks, shopping, taking medications, managing money (e.g. paying bills).

4 Note that this question was only asked if a person claimed to have received some form of home care during the pandemic.

5 We opted for categorising the responses this way to ensure all categories are substantially populated.

6 In wave 2 of the pandemic survey, the question is framed differently, precluding construction of a comparable indicator.

7 Adjusted for the number of people in the household using the OECD equivalence scale.

8 Note that respondents’ characteristics are measured at wave 9 of the survey and hence do not change in COVID waves surveys.

9 Note that we were able to construct reasonably comparable variables only for the pre-pandemic period and COVID wave 1, so COVID wave 2 is excluded from this analysis.

10 We note that this model does not pass the formal requirements of the independence of irrelevant alternatives assumptions. Appendix B explains why, and shows that alternative specifications which pass formal tests yield very similar results.

11 The numbers are inferred from Table 1 by deducting the share of respondents with no home care needs from unity.

## References

[b1] Addario G., Dangerfield P., Hussey D., Pacchioti B., Wood M. (2020). Adapting fieldwork during the COVID-19 outbreak: A methodological overview of the ELSA COVID-19 substudy (wave 1). NatCen.

[b2] Bergeot J., Jusot F. (2024). How did unmet care needs during the pandemic affect health outcomes of older European individuals?. Econ. Hum. Biol..

[b3] Bergmann M., Wagner M. (2021). The impact of COVID-19 on informal caregiving and care receiving across Europe during the first phase of the pandemic. Front. Public Heal..

[b4] Bottery S., Mallorie S. (2024). https://www.kingsfund.org.uk/insight-and-analysis/long-reads/social-care-360.

[b5] Bradshaw J., McLachlan G. (1972). Problems and Progress in Medical Care: Essays on Current Research.

[b6] Chen S., Jones L.A., Jiang S., Jin H., Dong D., Chen X., Wang D., Zhang Y., Xiang L., Zhu A. (2022). Difficulty and help with activities of daily living among older adults living alone during the COVID-19 pandemic: A multi-country population-based study. BMC Geriatr..

[b7] Di Novi C., Martini G., Sturaro C. (2023). The impact of informal and formal care disruption on older adults’ psychological distress during the COVID-19 pandemic in UK. Econ. Hum. Biol..

[b8] Dunatchik A., Icardi R., Blake M. (2019). Predicting unmet need for social care. J. Long-Term Care.

[b9] Evandrou M., Falkingham J., Qin M., Vlachantoni A. (2020).

[b10] Hu B., Cartagena-Farias J., Brimblecombe N., Jadoolal S., Wittenberg R. (2023). Projected costs of informal care for older people in England. Eur. J. Heal. Econ..

[b11] Jarling A., Rydström I., Fransson E.I., Nyström M., Dalheim-Englund A.-C., Ernsth Bravell M. (2022). Relationships first: Formal and informal home care of older adults in Sweden. Health Soc. Care Community.

[b12] Kjær A.A., Siren A. (2020). Formal and informal care: Trajectories of home care use among Danish older adults. Ageing Soc..

[b13] Lera J., Pascual-Sáez M., Cantarero-Prieto D. (2021). Socioeconomic inequality in the use of long-term care among European older adults: An empirical approach using the share survey. Int. J. Environ. Res. Public Heal..

[b14] Lyu J.Y., Hu B., Wittenberg R., King D. (2023). The relationships between informal and formal social care for older people in England: A comparison before and after the Care Act 2014. J. Aging Soc. Policy.

[b15] NHS England J.Y. (2020). https://www.england.nhs.uk/2020/08/covid-friendly-cancer-treatments/.

[b16] OECD J.Y. (2018). https://www.oecd-ilibrary.org/content/component/9789264085107-graph7-en.

[b17] OECD J.Y. (2023). https://www.oecd-ilibrary.org/content/component/4fc2cb96-en.

[b18] Rodrigues R., Simmons C., Schmidt A.E., Steiber N. (2021). Care in times of COVID-19: The impact of the pandemic on informal caregiving in Austria. Eur. J. Ageing.

[b19] Saloniki E.-C., Nizalova O., Malisauskaite G., Forder J. (2024). The impact of formal care provision on informal care receipt for people over 75 in England. PLoS One.

[b20] Spiers G.F., Kunonga T.P., Stow D., Hall A., Kingston A., Williams O., Beyer F., Bower P., Craig D., Todd C. (2022). Factors associated with unmet need for support to maintain independence in later life: A systematic review of quantitative and qualitative evidence. Age Ageing.

[b21] Suanet B., Van Groenou M.B., Van Tilburg T. (2012). Informal and formal home-care use among older adults in Europe: Can cross-national differences be explained by societal context and composition?. Ageing Soc..

[b22] Topriceanu C.-C., Wong A., Moon J.C., Hughes A.D., Bann D., Chaturvedi N., Patalay P., Conti G., Captur G. (2021). Evaluating access to health and care services during lockdown by the COVID-19 survey in five UK national longitudinal studies. BMJ Open.

[b23] Tur-Sinai A., Bentur N., Fabbietti P., Lamura G. (2021). Impact of the outbreak of the COVID-19 pandemic on formal and informal care of community-dwelling older adults: Cross-national clustering of empirical evidence from 23 countries. Sustainability.

[b24] Vislapuu M., Angeles R.C., Berge L.I., Kjerstad E., Gedde M.H., Husebo B.S. (2021). The consequences of COVID-19 lockdown for formal and informal resource utilization among home-dwelling people with dementia: Results from the prospective PAN. DEM study. BMC Health Serv. Res..

[b25] Vlachantoni A., Shaw R., Willis R., Evandrou M., Falkingham J., Luff R. (2011). Measuring unmet need for social care amongst older people. Popul. Trends.

[b26] Zigante V., Fernandez J.-L., Mazzotta F. (2021). Changes in the balance between formal and informal care supply in England between 2001 and 2011: Evidence from census data. Heal. Econ. Policy Law.

